# A Mixed-Methods Study of Risk Factors and Experiences of Health Care Workers Tested for the Novel Coronavirus in Canada

**DOI:** 10.1097/JOM.0000000000002614

**Published:** 2022-06-14

**Authors:** Arnold Ikedichi Okpani, Stephen Barker, Karen Lockhart, Jennifer Grant, Jorge Andrés Delgado-Ron, Muzimkhulu Zungu, Nisha Naicker, Rodney Ehrlich, Annalee Yassi

**Affiliations:** From the School of Population and Public Health (SPPH), University of British Columbia, Vancouver, British Columbia, Canada (Dr Okpani, Mr Barker, Ms Lockhart, Dr Delgado-Ron, Ms Yassi); Physician Occupational Safety and Health, Vancouver Coastal Health, Vancouver, British Columbia, Canada (Ms Yassi, Dr Grant, Ms Yassi); Department of Pathology and Laboratory Medicine, Vancouver Coastal Health, Vancouver, British Columbia, Canada (Dr Grant); National Institute for Occupational Health, a Division of National Health Laboratory Service, Johannesburg, South Africa (Dr Zungu); School of Health Systems and Public Health, University of Pretoria, Pretoria, South Africa (Dr Zungu); Department of Environmental Health, University of Johannesburg, Johannesburg, South Africa (Dr Naicker); Division of Occupational Medicine, School of Public Health and Family Medicine, University of Cape Town, Cape Town, South Africa (Dr Ehrlich).

**Keywords:** COVID-19, epidemiology, health personnel, infections, occupational health, workplace

## Abstract

The aims of this study were to investigate occupational and non–work-related risk factors of coronavirus disease 2019 among health care workers (HCWs) in Vancouver Coastal Health, British Columbia, Canada, and to examine how HCWs described their experiences.

**Methods:**

This was a matched case-control study using data from online and phone questionnaires with optional open-ended questions completed by HCWs who sought severe acute respiratory syndrome coronavirus 2 testing between March 2020 and March 2021. Conditional logistic regression and thematic analysis were utilized.

**Results:**

Providing direct care to coronavirus disease 2019 patients during the intermediate cohort period (adjusted odds ratio, 1.90; 95% confidence interval, 1.04 to 3.46) and community exposure to a known case in the late cohort period (adjusted odds ratio, 3.595%; confidence interval, 1.86 to 6.83) were associated with higher infection odds. Suboptimal communication, mental stress, and situations perceived as unsafe were common sources of dissatisfaction.

**Conclusions:**

Varying levels of risk between occupational groups call for wider targeting of infection prevention measures. Strategies for mitigating community exposure and supporting HCW resilience are required.

## Abstract 2

A reappraisal of mitigation strategies against occupational hazards is required to ensure that all health workers – not just those in perceived “high-risk” work environments - are protected. This will mean reducing community-level risks and ensuring that the same level of IPC measures, PPE training and supply is available when needed.

The severe acute respiratory syndrome coronavirus 2 (SARS-CoV-2) has had a devastating effect on the health and well-being of health care workers (HCWs). An estimated 152,888 HCWs had become infected worldwide, with 1413 dying of the coronavirus disease 2019 (COVID-19) by early May 2020.^1^ Infection, disease, and death among HCWs continued through the different phases of the pandemic. Health care workers also continue to experience new or worsening mental stress.^2–5^ The burden of infection has varied widely across jurisdictions, as has the contribution of known risk factors. In Canada, HCWs accounted for 19.4% of all detected cases between February and July 2020.^6^ By June 2021, that proportion declined to 6.8% of cases, with substantial variation across provinces—12.3% in Quebec, 5.5% in British Columbia (BC), and 4.4% in Ontario.

Some reports indicate that community, rather than the workplace exposure, is the main driver of SARS-CoV-2 infection among HCWs.^7,8^ Others have shown an association between provision of direct care to COVID-19 patients and elevated risk of infection.^9,10^ Further studies found that working in dedicated COVID-19 wards was associated with lower risk,^11,12^ likely attributable to better availability and use of personal protective equipment (PPE) in units considered at high risk for COVID-19.^13^

The inconsistency of the foregoing findings suggests contextual differences in the predictors of infection risk. The Vancouver Coastal Health (VCH) region of BC, Canada, is an example of a jurisdiction where HCW infections have been comparatively low because of infection prevention and control (IPC) and other mitigation strategies in health care.^14^ In addition, VCH implemented other strategies such as asymptomatic on-site testing following suspected or confirmed workplace exposure, dedicated test sites, and priority testing and vaccination for HCWs. The perception of HCWs of these interventions—in relation to their self-assessed sense of safety at work—has not been documented.

We therefore aimed to investigate the contribution of occupational and non–work-related factors to the risk of SARS-CoV-2 infection among HCWs in VCH and to explore the lived experience of HCWs who sought testing for SARS-CoV-2, including their perception of personal safety through the phases of the pandemic.

## MATERIALS AND METHODS

Vancouver Coastal Health is one of BC's five health authorities and provides care for 25% of the province's population.^15^ It serves as the referral region for advanced care. Individuals who attended a VCH coronavirus testing center between March 1, 2020, and March 31, 2021, and self-identified as HCWs were contacted by the health authority to inform them of a study investigating risk factors for COVID-19 among HCWs. Identification of eligible participants was made possible because all polymerase chain reaction test results at health facility and community test sites in BC were centrally reported. Those who agreed to be contacted were asked to complete a self-administered online questionnaire or to participate in a telephone interview. Each respondent had the option of providing free-text comments on their experience, sense of safety at work and in the community, or any other issues. Data collection took place between November 9, 2020, and June 30, 2021.

We used a mixed-methods approach to integrate qualitative data into a matched case-control study design. The embedded mixed-method approach^16^ allowed the authors to examine potential mechanisms that might explain the result of statistical models.

The study protocol was approved by the University of British Columbia Behavioral Research Ethics Board (H20-02517).

### Exposures and Variables

We collected data on participants' demographics, worksite, occupation, activities, and behavior at and outside of work in the 2 weeks preceding their test dates. We asked about the respondent's travel modes to work, exposure to known COVID-19 cases, and use of PPE. Respondents tested after December 15, 2020, were asked about vaccination (status and dates). If tested more than once, respondents were asked to provide the date of the test for which they had the clearest recall of their activities 2 weeks prior and to complete the questionnaire based on that recall period. With that date and their personal health number—which they supplied as part of the consent process—their test results were extracted from VCH laboratory records. The text of the electronic study questionnaire is included as Supplemental Digital Content 1, http://links.lww.com/JOM/B138.

Health care workers who tested positive for SARS-CoV-2 in a nucleic acid amplification test conducted on a nasal swab or mouth gargle specimen were included as cases. Health care workers with a negative result were included as controls. Cases and controls were matched without replacement only by the week of the test, in a 1:4 ratio. We excluded individuals with indeterminate or missing test results.

### Quantitative Analyses

The odds of a positive SARS-CoV-2 test were estimated for different occupational exposures. These included direct care to COVID-19 patients, exposure to patients' materials or body fluids, work in proximity (≤2 m for ≥15 minutes) with colleagues, work with a colleague who subsequently tested positive for SARS-CoV-2 within the 2 weeks before their own test, and difficulty accessing PPE or reusing PPE. We also estimated the odds of infection for HCWs in nonoccupational exposure settings: extended close contact (≤2 m for ≥15 minutes) with a known COVID-19–infected or symptomatic individual (fever, cough, runny nose, sore throat, shortness of breath), international travel, public transport, and general social interaction with individuals outside of work or home contacts.

We summarized respondents' characteristics using means, SD, and proportions, stratified by the outcome. Unadjusted logistic regression was used to estimate odds ratios (ORs) with 95% confidence intervals (CIs). Multivariable conditional logistic regression—adjusted for age, gender, race, occupation (where appropriate), and number of weeks since pandemic declared—was used to estimate adjusted ORs (aORs). Covariate selection was informed by the results of previous studies.^10,12^

To assess effect modification by pandemic phase, we categorized respondents by test date into three cohorts: respondents tested between March 17, 2020 (date public health emergency declared in BC),^17^ and August 31, 2020 (date BC Provincial Health Officer signaled the start of a potential second wave),^18^ were included in the early cohort (EC). Those tested between September 1 and December 14, 2020, were included in the intermediate cohort (IC). Those who tested between December 15, 2020, when SARS-CoV-2 vaccination was introduced, and March 31, 2021 (study SARS-CoV-2 test eligibility end date), were included in the late cohort (LC).

All statistical analyses were conducted using R version 3.6.1 (R Foundation for Statistical Computing, Vienna, Austria).

### Qualitative Analysis

Deidentified free-text responses from the questionnaires were exported to a Microsoft Excel (Microsoft Inc, Redmond, WA) spreadsheet for coding and analysis. We first adopted a deductive approach by categorizing responses according to predefined categories. We then examined the data inductively to identify main themes that fit within the preset categories, adding as new categories main themes that did not fit. Subthemes were identified within each of the main themes in a constant comparative process. Initial coding was conducted by A.I.O., with 25% independently coded by J.A.D.-R. Disagreements were resolved by consensus.

## RESULTS

**FIGURE 1:**
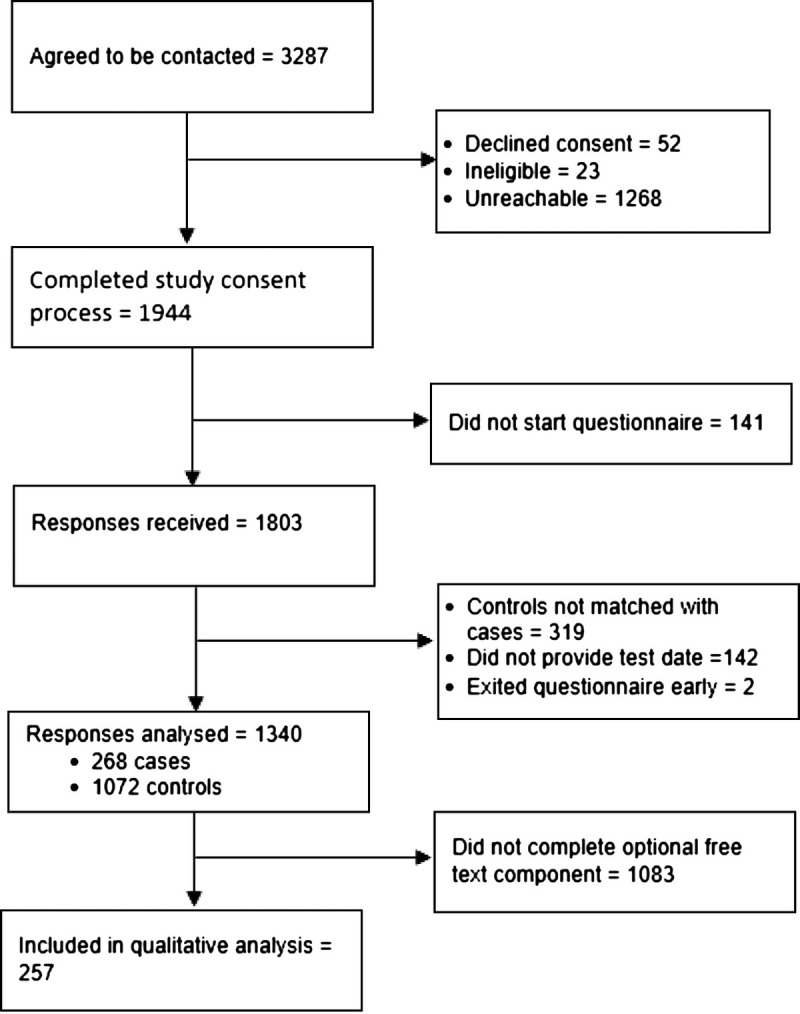
Study flow diagram.

We received 1659 responses to the structured component of the questionnaire (43.3% response rate). Of these, 268 were cases. With 1:4 matching, 1340 observations were included in the quantitative analysis (Fig. 1). We received free-text responses to the optional open-ended component of the questionnaire from 257 respondents. Sixty-one of the responses came from cases, and 196 were from controls. A chi-square test of independence between case status and provision of free-text responses indicated no association (*P* = 0.09).

Respondents' characteristics are shown in Table 1. Most respondents were female, residents of VCH, and Canadian citizens and had tertiary education. Most self-identified as Asian or non-Hispanic White. There were more acute and community care workers than long-term care (LTC) workers. There was a higher proportion of care aides/licensed practical nurses (LPNs) among the cases than other occupations.

**TABLE 1:** Characteristics of Study Participants

Characteristics	Cases (n = 268)	Controls (n = 1072)
Mean (SD) age, y	41.2 (12.9)	41.3 (11.8)
Gender		
Female	203 (75.7%)	816 (76.1%)
Male	61 (22.8%)	241 (25.5%)
Nonbinary	4 (1.5%)	10 (0.9%)
Prefer not to answer	0 (0.0%)	5 (0.5%)
Postal code of residence		
Vancouver Coastal Health	237 (88.4%)	949 (88.5%)
Fraser Health	26 (9.7%)	114 (10.6%)
Other	5 (1.9%)	9 (0.9%)
Status in Canada		
Citizen	228 (85.1%)	972 (90.7%)
Permanent resident	30 (11.2%)	86 (8.0%)
Visitor	2 (0.7%)	5 (0.5%)
Prefer not to answer	8 (3.0%)	9 (0.8%)
Indigenous person		
Yes	10 (3.7%)	27 (2.5%)
No	257 (95.9%)	1034 (96.5%)
Prefer not to answer	1 (0.4%)	11 (1.0%)
Race		
Asian	102 (38.1%)	344 (32.1%)
Non-Hispanic White	128 (47.8%)	587 (54.8%)
Indigenous	7 (2.6%)	19 (1.8%)
Other	23 (8.6%)	87(8.1%)
Prefer not to answer	8 (3.0%)	35 (3.3%)
Education level		
Secondary or less	24 (9.0%)	78 (7.3%)
Tertiary/university	147 (55.1%)	602 (56.2%)
Postgraduate	84 (31.5%)	355 (33.1%)
Prefer not to answer	12 (4.5%)	37 (3.5%)
Worksite		
Acute care	101 (37.7%)	470 (43.8%)
Community	133 (49.6%)	473 (44.1%)
Long-term care	34 (12.7%)	129 (12.0%)
Underlying health condition		
Yes	68 (25.4%)	257 (24.0%)
No/unknown	173 (64.6%)	710 (66.2%)
No answer	27 (10.1%)	105 (9.8%)
Smoking status		
Current smoker	5 (1.9%)	49 (4.6%)
Past smoker	47 (17.5%)	165 (15.4%)
Never smoked	189 (70.5%)	753 (70.2%)
No answer	27 (10.1%)	105 (9.8%)
Occupation		
Registered nurses	61 (22.8%)	263 (24.5%)
Care aides/licensed practical nurses	35 (13.1%)	66 (6.2%)
Administration	41 (15.3%)	152 (14.2%)
Allied health	87 (32.5%)	380 (35.4%)
Medical staff	27 (10.1%)	139 (13.0%)
Support staff	9 (3.4%)	36 (3.4%)
Other/unknown	8 (3.0%)	36 (3.4%)

SARS-CoV-2, severe acute respiratory syndrome coronavirus 2.

Assessing the relationship between work exposure and SARS-CoV-2–positive test among health care workers in Vancouver Coastal Health (March 2020–March 2021).

Analysis of the unstructured interview indicated that the framing of HCW experiences reflected the phase of the pandemic during which they sought testing. Main themes were lack of clarity and consistency of information—about when to get tested, isolation, contact tracing, test locations, and sample collection methods; perceived threats to personal safety at work and in the community; and anxiety about suspected exposure sources.

### Workplace Exposures

As shown in Table 2, direct patient care in the IC (aOR, 1.90; 95% CI, 1.04 to 3.46) and having more than 50 COVID-19 patient care encounters in the LC (aOR, 6.78; 95% CI, 1.05 to 43.84) were associated with infection. Contact with patients' materials, being present for an AGP, and worksite were not associated with infection in any of the cohorts. In contrast, in the pooled analysis (Supplemental Digital Content 2, http://links.lww.com/JOM/B139), HCWs who reported difficulty getting PPE had 1.84 times the odds of infection compared with those who did not report difficulty (95% CI, 1.07 to 3.17). Reuse of PPE was not significantly associated with infection.

**TABLE 2:** Test Date Cohort-Stratified OR for the Relationship Between Work Exposure and SARS-CoV-2–Positive Test Among Health Care Workers in Vancouver Coastal Health (March 2020–March 2021)

Variable	Early Cohort Adjusted OR* n: 31 Cases, 137 Controls			Intermediate Cohort Adjusted OR* n: 101 Cases, 420 Controls			Late Cohort Adjusted OR† n: 136 Cases, 515 Controls		
	n1	n2	OR (95% CI)	n1	n2	OR (95% CI)	n1	n2	OR (95% CI)
Direct COVID-19 patient care role									
No	21	85	1 (ref)	59	296	1 (ref)	105	372	1 (ref)
Yes	10	52	0.39 (0.11–1.43)	40	123	1.90 (1.04–3.46)	31	142	1.14 (0.59–2.19)
Close contact with known COVID-19 patient									
No/unknown	22	102	1 (ref)	71	331	1 (ref)	111	411	1 (ref)
<10 times	7	15	2.52 (0.51–12.38)	4	37	0.64 (0.20–2.04)	10	26	1.84 (0.55–6.14)
10–50 times	1	4	2.84 (0.07–110.10)	11	13	2.27 (0.85–6.04)	6	21	1.67 (0.43–6.54)
>50 times	1	3	5.31 (0.04–728.55)	4	5	3.83 (0.70–20.95)	3	7	6.78 (1.05–43.84)
Direct contact with patient's materials									
No/unknown	15	79	1 (ref)	41	219	1 (ref)	77	257	1 (ref)
Yes	16	44	1.32 (0.40–4.40)	49	166	1.28 (0.73–2.23)	52	207	0.80 (0.48–1.36)
Present for aerosol generating procedure on COVID-19 patient									
No	29	111	1 (ref)	82	359	1 (ref)	122	439	1 (ref)
Yes	1	5	0.39 (0.02–8.36)	5	11	2.37 (0.62–9.01)	5	19	1.80 (0.36–9.06)
Unknown	1	8	NA	3	16	0.59 (0.15–2.27)	3	7	3.08 (0.32–29.63)
Worksite									
Acute care	16	62	1 (ref)	42	193	1 (ref)	43	215	1 (ref)
Community	11	45	0.98 (0.29–3.35)	42	178	1.35 (0.72–2.53)	80	250	1.01 (0.58–1.76)
Long-term care	4	30	0.88 (0.17–4.45)	17	49	1.57 (0.68–3.62)	13	50	1.06 (0.37–3.01)
Extended close contact with coworker (within 2 m for ≥15 min)									
No	4	36	1 (ref)	41	141	1 (ref)	67	168	1 (ref)
Yes	27	98	3.51 (0.77–16.12)	54	261	0.51 (0.29–0.89)	65	320	0.69 (0.42–1.12)
Made aware that close-worker contact tested positive afterward									
No close contact with coworker	4	36	1 (ref)	41	141	1 (ref)	67	168	1 (ref)
Contact, coworker not positive	22	75	4.18 (0.82–21.24)	47	201	0.58 (0.33–1.04)	57	262	0.74 (0.44–1.22)
Contact with positive coworker	5	23	2.49 (0.38–16.32)	6	58	0.28 (0.10–0.78)	8	52	0.40 (0.14–1.19)
Work involves contact with patient's materials, belongings, or equipment									
No	2	9	1 (ref)	21	84	1 (ref)	37	112	1 (ref)
Yes	9	30	2.21 (0.26–18.44)	61	277	0.63 (0.31–1.24)	95	369	1.13 (0.62–2.08)
No response	20	98	1.09 (0.15–8.10)	19	59	1.29 (0.49–3.41)	4	34	1.00 (0.98–1.03)
Experienced difficulty getting any PPE									
No	19	95	1 (ref)	64	279	1 (ref)	84	339	1 (ref)
Yes	10	20	1.09 (0.29–4.07)	6	25	0.69 (0.19–2.56)	9	16	2.43 (0.78–7.59)
Reused PPE on account of an inadequate supply									
No	17	89	1 (ref)	55	255	1 (ref)	84	302	1 (ref)
Yes	12	26	2.32 (0.60–8.89)	15	49	0.90 (0.39–2.11)	9	53	0.79 (0.30–2.12)

CI, confidence interval; COVID-19, coronavirus disease 2019; n1, number of cases; n2, number of controls; OR, odds ratio; PPE, personal protective equipment; ref, reference group; SARS-CoV-2, severe acute respiratory syndrome coronavirus 2.

*Adjusted for categorical age, gender, race, occupation, and number of weeks since pandemic declared.

†Adjusted for categorical age, gender, race, occupation, number of weeks since pandemic declared, and vaccination status.

In the free text responses, several respondents indicated that their employer had worked hard to provide a safe work environment in the face of supply constraints. An IC care aide thought that:

VCH has really gone above and beyond, getting as much supplies for us as possible. Unfortunately, there is just more demand than what can be supplied. I genuinely feel as though VCH [has] the best intentions for our safety.

The odds of infection were significantly lower among HCWs who worked in close proximity with a colleague in the IC (aOR, 0.51; 95% CI, 0.29 to 0.89; Table 2). Similarly, in the IC, the odds of infection were lower among HCWs who worked with a colleague with a positive test in the 2 weeks before the respondents' own test compared with those who did not (aOR, 0.28; 95% CI, 0.10 to 0.78). In the pooled analysis, the OR of positive SARS-CoV-2 comparing HCWs who worked in close proximity with colleagues with those who did not was 0.73 (95% CI, 0.54 to 0.98) (Supplemental Digital Content 2, http://links.lww.com/JOM/B139). The same lower odds were present for those who worked with a colleague who tested positive in the 2 weeks before the respondents' test (aOR, 0.39; 95% CI, 0.23 to 0.68). An LC registered nurse (RN) offered a potential explanation for this finding:

[I had been] tested three times, first two times were done due to exposure for a shift, supposedly I possibly worked during period where COVID-positive coworker worked, so I was asked to be tested twice. I was never symptomatic.

As shown in Table 3, comparing infection risk among occupational groups indicated that a difference in infection risk was only present in the IC, with all other occupational groups having higher odds of infection compared with medical staff: care aides/LPNs having 15 times the odds of infection (95% CI, 3.70 to 61.45). In the pooled analysis, care aides/LPNs had 2.73 times the odds of infection compared with medical staff (95% CI, 1.47 to 5.14). Belonging to other occupational groups was no longer associated with elevated risk. In post hoc analysis, adjusting for education and home postcode as proxies for socioeconomic status (SES) increased the odds of infection to 2.91, comparing care aides/LPNs with medical staff, Supplemental Digital Content 3, http://links.lww.com/JOM/B140.

**TABLE 3:** Test Date Cohort-Stratified OR for the Relationship Between Occupation and SARS-CoV-2–Positive Test Among Health Care Workers in Vancouver Coastal Health Region (March 2020–March 2021)

Variable	Early Cohort Adjusted OR* n = 31 Cases, 137 Controls			Intermediate Cohort Adjusted OR* n = 101 Cases, 420 Controls			Late Cohort Adjusted OR† n = 136 Cases, 515 Controls		
	n1	n2	OR (95% CI)	n1	n2	OR (95% CI)	n1	n2	OR (95% CI)
Medical staff	8	31	1 (ref)	4	56	1 (ref)	15	52	1 (ref)
Care aides/licensed practical nurses	3	17	0.84 (0.10–7.22)	17	21	15.08 (3.70–61.45)	15	28	1.45 (0.47–4.40)
Administration	4	22	0.44 (0.07–2.98)	12	59	3.68 (0.95–14.28)	25	71	0.81 (0.31–2.10)
Allied health	10	36	1.20 (0.25–5.82)	25	148	3.79 (1.07–13.51)	52	196	0.61 (0.26–1.41)
Registered nurses	4	22	1.76 (0.27–11.56)	36	109	5.22 (1.46–18.71)	21	132	0.58 (0.23–1.51)
Support staff	2	7	5.59 (0.34–90.67)	4	14	5.84 (1.04–32.75)	3	15	0.58 (0.11–2.94)
Other/unknown	0	2	NA	3	13	4.69 (0.75–29.52)	5	21	0.59 (0.15–2.32)

CI, confidence interval; n1, number of cases; n2, number of controls; NA, not applicable; OR, odds ratio; ref, reference group; SARS-CoV-2, severe acute respiratory syndrome coronavirus 2.

*Adjusted for categorical age, gender, race, and number of weeks since pandemic declared.

†Adjusted for categorical age, gender, race, number of weeks since pandemic declared, and vaccination status.

Respondents in work areas that were not considered “high risk” recounted feeling unsafe as they were not prioritized to receive PPE supplies and training especially when supplies were limited. An EC reception clerk mentioned that:

“In March and April, there were not enough masks for staff, and everyone thinks clerical workers [do not] touch patients and [they] do not need to wear mask.” An EC cohort administration clerk noted the same concern: “The clinical resource nurse we had refused to make available masks, hand sanitizer, and face shields to clerical staff prior to when I was tested. Even though equipment was available, we had to talk to [them] every time we needed a new mask.” An EC administration worker stated that “Always remember clerical staff are as important as nurses, and that even though we may not physically interact with clients—like nurses do—we are still in contact with them. We are often forgotten when it comes to training [on] how to properly put on PPE and with masks fittings.”

### Community Exposure

Table 4 shows that exposure to a known COVID-19 case outside of work was significantly associated with infection in the LC (aOR, 3.51; 95% CI, 1.86 to 6.63). The estimated OR of infection associated with social interaction was 3.50 (95% CI, 1.12 to 10.90) for those who had social contact on some days in the IC compared with those who reported no social interaction. Results of the pooled analysis (Supplemental Digital Content 4, http://links.lww.com/JOM/B141) show that exposure to a known COVID-19 case outside of work was associated with infection (aOR, 2.45; 95% CI, 1.67 to 3.59). Similarly, exposure to an individual with symptoms related to COVID-19 was associated with infection (aOR, 1.53; 95% CI, 1.07 to 2.21). International travel and use of public transport were not associated with infection in the stratified and pooled analysis.

**TABLE 4:** Test Date-Stratified OR for the Relationship Between Non–Work-Related Risk Factors and SARS-CoV-2–Positive Test Among Health Care Workers in Vancouver Coastal Health (March 2020–March 2021)

Variable	Early Cohort Adjusted OR* n = 31 Cases, 137 Controls			Intermediate Cohort Adjusted OR* n = 101 Cases, 420 Controls			Late Cohort Adjusted OR† n = 136 Cases, 515 Controls		
	n1	n2	OR (95% CI)	n1	n2	OR (95% CI)	n1	n2	OR (95% CI)
Extended close contact with a person or persons known to have been diagnosed with COVID-19 (outside occupational duty)									
No	20	96	1 (ref)	63	310	1 (ref)	76	370	1 (ref)
Yes	3	13	1.60 (0.17–14.78)	17	35	1.75 (0.82–3.73)	34	65	3.51 (1.86–6.63)
Extended close contact with a person with COVID-19 symptoms (outside occupational duty)									
No	19	82	1 (ref)	65	275	1 (ref)	77	351	1 (ref)
Yes	4	25	1.48 (0.27–8.00)	19	60	1.44 (0.73–2.87)	28	72	1.62 (0.83–3.15)
Return from international travel									
No	31	129	1 (ref)	93	404	1 (ref)	133	491	1 (ref)
Yes	0	5	NA	2	1	NA	1	2	1.93 (0.10–38.13)
Use of public transport									
Did not use public transport	21	98	1 (ref)	79	333	1 (ref)	100	394	1 (ref)
Few days (≤3 d)	5	13	2.93 (0.39–21.88)	9	31	0.99 (0.40–2.47)	13	44	1.51 (0.67–3.45)
Some days (4–7 d)	3	5	2.21 (0.29–16.53)	2	14	1.15 (0.21–6.40)	9	23	1.65 (0.57–4.74)
Most days (≥8 d)	2	18	0.11 (0.01–1.34)	5	26	0.65 (0.18–2.30)	11	31	1.44 (0.53–3.94)
Social interactions with individuals outside of work or home									
Did not have any such social interactions	18	63	1 (ref)	51	259	1 (ref)	83	274	1 (ref)
Few days (≤3 d)	9	55	0.24 (0.07–0.84)	34	123	1.67 (0.93–2.97)	40	170	0.95 (0.56–1.64)
Some days (4–7 d)	3	13	0.24 (0.03–2.15)	8	15	3.50 (1.12–10.90)	6	35	0.60 (0.20–1.76)
Most days (≥8 d)	1	3	0.44 (0.01–19.31)	2	6	2.09 (0.24–18.09)	4	11	1.53 (0.37–6.31)

CI, confidence interval; COVID-19, coronavirus disease 2019; n1, number of cases; n2, number of controls; NA, not applicable (there were too few responses due to travel restrictions); OR, odds ratio; ref, reference group; SARS-CoV-2, severe acute respiratory syndrome coronavirus 2.

*Adjusted for categorical age, gender, race, and number of weeks since pandemic declared.

†Adjusted for categorical age, gender, race, number of weeks since pandemic declared, and vaccination status.

Many HCWs considered themselves safer at work (even when caring for known COVID-19 patients) than in the community. The reason cited was the sense that workplace policies and PPE were keeping them safe, whereas they had no control over other people's choices in the community. An LC RN explained that:

[Our] workplace has taken many precautions to keep us safe. Masks and measures are in place, and most people have had at least one dose of vaccine in the office. I do not think it is safe in the community for those unvaccinated or not exposed to virus.

An LC social worker had the same view:

[I] Feel safer while at work in hospital, even going on to COVID unit and speaking with patients who are awaiting second negative test, than I do in community (especially on a bus).

Some participants reported feeling safe in the community because of their unique locations or a sense of control they have over where they can be. An LC admin staff member reported: “We are in a remote community and are keeping to ourselves. Even at work, we socially distance and sanitize frequently.” AN LC RN mentioned that: “I find it safer in the community because I can control it. If anywhere is busy, I will leave. It is different in the workplace setting.”

### Concerns on Getting Tested, Handling Test Outcomes, and Working During the Pandemic

Respondents recounted concerns related to having to deal with rapidly changing policies and protocols, difficulties in accessing tests and results, feelings of stigma, mental stress, and other forms of hardship.

As stated by a LC physical therapist:

My [spouse] and myself kept getting potentially contradictory instructions from multiple different nurses and public health officers, who would also routinely explain that they would need to consult a doctor before they could make further recommendations… our isolation instructions were very poorly communicated. My [spouse] ended up getting 5 COVID tests that were all negative over a 1-week period, then an antibody test that further confirmed that [they] had not had COVID.

An EC RN noted that “the policy at my worksite is unclear and different depending on which manager you speak to.”

The descriptions of the test experience changed by test site. On-site testing in health facilities and dedicated lanes for HCWs in community test centers were positively received. Health care workers who went to drive-through test sites recounted shorter wait times and fewer privacy concerns than those who went to walk-in sites. An IC unit clerk recounted:

Great having a separate lineup for health care workers. Very short time spent waiting in the car via drive-through. Staff were knowledgeable and quick.

This contrasted with the experience of an IC RN:

Very little privacy provided at testing site. Personal information was yelled back and forth with other non–health care people around. No attempt was made to make this less open.

Some respondents felt they were treated with disdain after they tested positive. Yet, others described feeling shame and not wanting to let others know they tested positive. The mental stress of working in the pandemic was also raised. An LC RN recounted their unpleasant experience:

It's a cold and sad experience and I felt that I was treated as a burden on the system. One person who did the check-in was nasty to me. It was clear that the staff were afraid of me. I certainly never want to go through it again.

An LC community support worker shared a similar experience:

I felt extreme stress and shame. All my coworkers found out I had COVID, and some were great about it, but some were not. One thing I feel moving forward is maybe more support for people who are isolating in their rooms. We should also remind people who test positive, it's not their fault.

Recounting the mental stress of isolation, an IC RN stated:

Because of my risk of exposure at work and my underlying condition, I strictly limit my contacts outside of work to my household only and have been doing this the entire pandemic. This is taking a significant toll on my mental health, and on my family as I am unable to be available for support to my elderly and unwell parents.

Some respondents indicated they decided to change jobs, worksites, or careers due to anxiety about health and safety. An IC RN recounted their experience before leaving nursing:

Awful situation in LTC facilities. Very limited PPE available. No official announcement on COVID outbreak at facility, heard about it through grapevine. No PPE available at the beginning, letting families in to visit, mixing COVID patients with other patients for dining. Completely run down and very stressed. Subsequently left the role and nursing and now working in another sector.

An IC social worker described a similar experience:

I worked at a clinic [where] I felt very unsafe there. The IPAC practices were not good. They let patients in without masks. They had no isolation room. [Three] Workers got COVID at that site, including me. I have resigned from that site.

## DISCUSSION

In this study of HCWs in the VCH region, we found that certain occupational factors, alongside community exposures, were associated with a positive SARS-CoV-2 test. The phase of the pandemic during which the HCWs got tested was relevant in determining their infection risk. Whereas direct care to COVID-19 patients was a significant factor in the second wave of the pandemic, contact with patients' materials, worksite, and difficulty obtaining PPE were not associated with infection. Whereas all occupational groups had a higher risk of infection compared with medical staff in the second wave, there was no difference between occupations in the other phases of the pandemic covered by our study. Exposure to a known case of COVID-19 in the community and having social interaction on some days were associated with infection at different phases of the pandemic in our study population.

Our finding of an association between direct COVID-19 patient care and infection among HCWs tested in the IC period of this study is consistent with the widely reported risk of occupational infection among HCWs, particularly in the earlier phases of the pandemic.^9,10,19^ The significant role of community exposure in HCW infections in this population is consistent with our previous finding that that rates in this group followed very closely the trend in the background population.^14^ A spike in HCW infections reported at the start of the pandemic quickly settled to mirror the picture in the community as workplace mitigation strategies improved. This finding from a 14-month surveillance study from our group^14^ and a follow-up study at 20 months^20^ reinforced the importance of measures such as PPE, point-of-care risk assessment, contact tracing, and IPC in keeping HCWs safe.

The foregoing is supported by the finding in the stratified analyses of non–work-related risk factors that close contact with a known case in the community in the late phase was associated with infection. By the end of the intermediate phase of the of the study period, the infection incidence rate among the VCH health care workforce dropped below community rates as reported in the surveillance study. This is undoubtedly related to the prioritization for vaccination of HCWs, especially those in high-risk settings. Infection incidence rates remained below the rates in the background community until the end of our study data collection period. Similarly, Jacob and colleagues,^8^ in a cross-sectional study that included HCWs across three states in the United States, reported that HCW infection was associated with COVID-19 incidence rates in their background communities. The findings from this case-control study, taken together with the results of the analysis of incidence rates of the entire cohort relative to background population, provide evidence of the importance of implementing strong infection prevention and public health measures to protect HCWs.

Working in LTC as opposed to acute care has been identified as being associated with infection in other studies (including our previous study). We did not find a significantly higher risk in LTC after accounting for occupation. However, we found that care aides/LPNs who work mostly in LTC had the highest risk of infection compared with medical staff. We could not conclude that this difference in risk was attributable to SES as we found an even stronger association when we accounted for education and home postcode as proxies for SES in the analysis. Residual confounding by SES remains a possibility, however, as other studies have indicated that socioeconomic factors can be important predictors of SARS-CoV-2 infection rates.^7,21^ As such, it is arguably all the more important to protect these HCWs from occupational exposure, to diminish their overall risk and reduce the risks they may bring into already more vulnerable community settings.

Our finding of a much lower risk of infection among HCWs who reported working in proximity with infected colleagues could result from the higher test frequency following exposure, leading to overrepresentation of such contacts among the controls. Public health protocols in BC promoted comprehensive contact tracing and a very low testing threshold—including asymptomatic testing—for HCWs exposed at work. The finding could also be partly attributable to better attention to, and availability of, PPE among HCWs in patient-facing settings. The foregoing agree with the findings of a rapid review of workplace policies useful in preventing COVID-19.^22^ Furthermore, HCWs in settings with high COVID-19 transmission were prioritized for vaccination in BC at a time when vaccines were not widely available.^23^

A further notable finding in the stratified analyses was the higher ORs relative to medical staff for care aides/LPNs, allied health personnel, RNs, and support staff only in the IC. This finding is unsurprising as the IC period was also a time of high community transmission before vaccines became available, and VCH medical staff had been shown to have the lowest rates of any occupational group in the health authority.^14^ Overall, however, findings in the stratified analysis were imprecise because of low case numbers in each subgroup, limiting any conclusion about trends. The low case numbers particularly relate to the first phase of the pandemic and our EC, from which we were able to recruit only 31 cases, compared with 100 and 136 for the IC and LC, respectively. This was partly due to relatively low VCH HCW case numbers for that period and the fact that our data collection began 9 weeks after the end of the EC period.

The experiences of mental stress and stigma reported by our study participants are consistent with findings from previous studies.^24–28^ As much as 75% of Canadian HCWs involved in COVID-19 direct care reported that their mental health deteriorated since the start of the pandemic,^5^ and a third of BC HCWs interviewed in a recent survey said they were considering quitting within the next 2 years.^29^ Not only has stigmatization of HCWs by certain members of the community been a problem through the pandemic,^30^ but so has stigmatization by fellow HCWs of colleagues who tested positive.^31^

In addition, the relationships between mental health, workplace stress, and adherence to infection control measures have long been known. For example, a study by Colindres and colleagues^32^ reported that not only was effort-reward imbalance in the health care workplace a predictor of burnout, but also that burnout was a negative unique incremental predictor of nurses' self-reported adherence with infection control measures. The reports of mental stress received in this study underline the multidirectional links between acquiring an occupational infection and workplace psychosocial well-being. This finding further emphasizes the need for specifically targeted strategies to protect HCWs from psychosocial hazards for its own sake but also as part of strategies to control biological hazards during a pandemic. As the pandemic drags on and new waves of infection continue to strain health services, measures to promote the resilience of weary HCWs are urgently needed. Research into strategies that can promote HCW mental and physical safety at work as well as beyond the workplace is needed.

Acquiring SARS-CoV-2 infection in the workplace is not inevitable. What is required is a reappraisal of the approach to implementing mitigation strategies to ensure that all HCWs—not only those in perceived “high-risk” work environments—are equally protected. The higher rate of infection among care aides/LPNs and HCWs who had difficulty getting PPE calls for better attention to ensuring a universal precautions approach throughout the health care sector. This will mean ensuring that the same levels of IPC measures, PPE training, and supply are available when needed to HCWs regardless of their occupational stature or setting in which they work.

### Limitations

Respondents self-selected into the study. Consequently, participants could be systematically different from HCWs who chose not to participate. Our findings, however, are consistent with the result of our group's previous study^14^ among VCH HCWs who comprised more than 80% of the respondents in this study. Second, in case-control studies, there is potential of differential recall between cases and controls. We would urge a cautious interpretation of our findings as they do not imply certainty about infection source. The method of generation of qualitative data we adopted may have precluded full in-depth interviews. That, however, was not a major objective of this study, and we did not set out to obtain qualitative interviews from all 1340 respondents. A more rigorous exploration of the themes generated would require a separate study.

## Supplementary Material

SUPPLEMENTARY MATERIAL
