# Supplementary material for: A Mixed-Methods Study of Risk Factors and Experiences of Health Care Workers Tested for the Novel Coronavirus in Canada
Source: J Occup Environ Med. 2022 Jun 14;64(9):e559–66. doi: 10.1097/JOM.0000000000002614 (PMC9426315; doi:10.1097/JOM.0000000000002614)
Supplement: SUPPLEMENTARY MATERIAL [file joem-64-e559-s002.docx]

**Supplemental Digital Content 2**

**Pooled odds ratio for the relationship between work exposure and SARS-CoV-2 positive test among healthcare workers in Vancouver Coastal Health (March 2020-March 2021)**

| **Variable** | **Cases**  **(n)** | **Controls (n)** | **Crude OR (95% CI)** | **Adjusted OR† (95% CI) n: 268 cases, 1072 controls** |
| --- | --- | --- | --- | --- |
| Direct COVID-19 patient care role |  |  |  |  |
| *No* | 185 | 753 | 1 (ref) | 1(ref) |
| *Yes* | 81 | 317 | 1.04(0.78 - 1.39) | 1.05(0.76 – 1.45) |
| Close contact with known COVID-19 patient |  |  |  |  |
| *No / unknown* | 204 | 844 | 1(ref) | 1(ref) |
| *< 10 times* | 21 | 78 | 1.11(0.66 - 1.81) | 1.15(0.68 – 1.96) |
| *10 - 50 times* | 18 | 38 | 1.96(1.07 – 3.45) | 1.67(0.91 – 3.07) |
| *> 50 times* | 8 | 15 | 2.20(0.88 – 5.15) | 2.12(0.81 – 5.53) |
| Direct contact with patient’s materials |  |  |  |  |
| *No / unknown* | 133 | 555 | 1(ref) | 1(ref) |
| *Yes* | 117 | 417 | 1.17(0.89 - 1.55) | 1.11(0.82 – 1.50) |
| Present for aerosol generating procedure on COVID-19 patient |  |  |  |  |
| *No* | 233 | 909 | 1(ref) | 1(ref) |
| *Yes* | 11 | 35 | 1.22(0.59 – 2.37) | 1.19(0.59 – 2.43) |
| *Unknown* | 7 | 31 | 0.88(0.35 – 1.91) | 0.78(0.34 – 1.81) |
| Work site |  |  |  |  |
| *Acute care* | 101 | 470 | 1(ref) | 1(ref) |
| *Community* | 133 | 473 | 1.31(0.98 – 1.74) | 1.29(0.94 – 1.77) |
| *Long-term care* | 34 | 129 | 1.22(0.78 – 1.87) | 0.85(0.51 – 1.41) |
| Extended close contact with coworker (within 2m for 15 minutes or more) |  |  |  |  |
| *No* | 112 | 345 | 1(ref) | 1(ref) |
| *Yes* | 146 | 679 | 0.66(0.50 - 0.87) | 0.65(0.49 – 0.87) |
| Made aware that close-worker contact tested positive afterwards |  |  |  |  |
| *No close contact with coworker* | 112 | 345 | 1(ref) | 1(ref) |
| *Contact, coworker not positive* | 126 | 538 | 0.72(0.54 – 0.96) | 0.73(0.54 – 0.98) |
| *Contact with positive coworker* | 19 | 133 | 0.44(0.25 – 0.73) | 0.39(0.23 – 0.68) |
| Work involves contact with patient’s materials, belongings, or equipment |  |  |  |  |
| *No* | 60 | 205 | 1(ref) | 1(ref) |
| *Yes* | 165 | 676 | 0.84(0.60 – 1.17) | 0.80(0.55 – 1.16) |
| *No response* | 43 | 191 | 0.77(0.49 – 1.19) | 0.70(0.40 – 1.19) |
| Experienced difficulty getting any PPE |  |  |  |  |
| *No* | 167 | 713 | 1(ref) | 1(ref) |
| *Yes* | 25 | 61 | 1.75(1.05 – 2.83) | 1.84(1.07 – 3.17) |
| Reused PPE on account of an inadequate supply |  |  |  |  |
| *No* | 156 | 646 | 1(ref) | 1(ref) |
| *Yes* | 36 | 128 | 1.16(0.76 – 1.74) | 1.18(0.77 – 1.80) |

† Adjusted for categorical age, gender, race, occupation, and number of weeks since pandemic declared.
ref=Reference group. PPE: Personal protective equipment.
