# Supplementary material for: A Mixed-Methods Study of Risk Factors and Experiences of Health Care Workers Tested for the Novel Coronavirus in Canada
Source: J Occup Environ Med. 2022 Jun 14;64(9):e559–66. doi: 10.1097/JOM.0000000000002614 (PMC9426315; doi:10.1097/JOM.0000000000002614)
Supplement: SUPPLEMENTARY MATERIAL [file joem-64-e559-s003.docx]

**Supplemental Digital Content 3**

**Pooled odds ratio for the relationship between occupation and SARS-CoV-2 positive test among healthcare workers in Vancouver Coastal Health region (March 2020-March 2021)**

| **Variable** | **Cases (n)** | **Controls (n)** | **Crude OR**  **(95% CI)** | **Adjusted OR† (95% CI) n: 268 cases, 1072 controls** | **Adjusted OR‡**  **(95% CI)**  **n: 268 cases, 1072 controls** |
| --- | --- | --- | --- | --- | --- |
| Medical staff | 27 | 139 | 1(ref) | 1(ref) | 1(ref) |
| Care-aides/licensed practical nurses | 35 | 66 | 2.73(1.53 – 4.92) | 2.75(1.47 – 5.14) | 2.91(1.47 – 5.77) |
| Administration | 41 | 152 | 1.39(0.82 – 2.40) | 1.36(0.77 – 2.38) | 1.49(0.81 – 2.73) |
| Allied health | 87 | 380 | 1.18(0.74 – 1.92) | 1.21(0.73 – 2.00) | 1.28(0.75 – 2.19) |
| Registered nurses | 61 | 263 | 1.19(0.73 – 1.99) | 1.22(0.72 – 2.09) | 1.37(0.75 – 2.50) |
| Support staff | 9 | 36 | 1.29(0.55 – 2.98) | 1.17(0.49 – 2.79) | 1.24(0.50 – 3.10) |
| Other/unknown | 8 | 36 | 1.14(048 – 2.73) | 1.00(0.40 – 2.48) | 1.08(0.42 – 2.75) |
| ref=Reference group. † Adjusted for categorical age, gender, race, and number of weeks since pandemic declared. ‡ Adjusted for categorical age, gender, race, number of weeks since pandemic declared, education and home postcode. OR: odds ratio | | | | | |
