# Supplementary material for: A Mixed-Methods Study of Risk Factors and Experiences of Health Care Workers Tested for the Novel Coronavirus in Canada
Source: J Occup Environ Med. 2022 Jun 14;64(9):e559–66. doi: 10.1097/JOM.0000000000002614 (PMC9426315; doi:10.1097/JOM.0000000000002614)
Supplement: SUPPLEMENTARY MATERIAL [file joem-64-e559-s004.docx]

**Supplemental Digital Content 4**

**Pooled odds ratio for the relationship between non-work-related risk factors and SARS-CoV-2 positive test among healthcare workers in Vancouver Coastal Health (March 2020-March 2021)**

| **Variable** | **Cases (n)** | **Controls (n)** | **Crude OR**  **(95% CI)** | **Adjusted OR† (95% CI) n: 268 cases, 1072 controls** |
| --- | --- | --- | --- | --- |
| Extended close contact with a person or persons known to have been diagnosed with COVID-19 (outside occupational duty) |  |  |  |  |
| *No* | 159 | 776 | 1(ref) | (1ref) |
| *Yes* | 54 | 113 | 2.33(1.61 – 3.35) | 2.45(1.67 – 3.59) |
| Extended close contact with a person with COVID-19 symptoms (outside occupational duty) |  |  |  |  |
| *No* | 161 | 708 | 1(ref) | 1(ref) |
| *Yes* | 51 | 157 | 1.43(0.99 – 2.03) | 1.53(1.07 – 2.21) |
| Return from international travel |  |  |  |  |
| *No* | 257 | 1024 | 1(ref) | 1(ref) |
| *Yes* | 3 | 8 | 1.49(0.33 – 5.20) | 1.56(0.38 – 6.41) |
| Use of public transport |  |  |  |  |
| *Did not use public transport* | 200 | 825 | 1(ref) | 1(ref) |
| *Few days (≤ 3 days)* | 27 | 88 | 1.26(0.79 – 1.97) | 1.20(0.75 – 1.91) |
| *Some days (4 - 7 days)* | 14 | 42 | 1.37(0.71 – 2.50) | 1.26(0.67 – 2.37) |
| *Most days (≥ 8 days)* | 18 | 75 | 0.99(0.56 – 1.66) | 0.92(0.53 – 1.60) |
| Social interactions with individuals outside of work or home |  |  |  |  |
| *Did not have any such social interactions* | 152 | 596 | 1(ref) | 1(ref) |
| *Few days (≤ 3 days)* | 83 | 348 | 0.93(0.69 – 1.26) | 0.94(0.69 – 1.28) |
| *Some days (4 - 7 days)* | 17 | 63 | 1.06(0.58 – 1.82) | 1.03(0.58 – 1.81) |
| *Most days (≥ 8 days)* | 7 | 20 | 1.37(0.53 – 3.16) | 1.36(0.55 – 3.33) |

† Adjusted for categorical age, gender, race, occupation, and number of weeks since pandemic declared. NA: Not applicable (there were too few responses due to travel restrictions). ref=Reference group. OR: odds ratio.
