# Supplementary material for: A Mixed-Methods Study of Risk Factors and Experiences of Health Care Workers Tested for the Novel Coronavirus in Canada
Source: J Occup Environ Med. 2022 Jun 14;64(9):e559–66. doi: 10.1097/JOM.0000000000002614 (PMC9426315; doi:10.1097/JOM.0000000000002614)
Supplement: Supplementary file 4 [file joem-64-e559-s005.docx]

Case Control Vancouver

Start of Block: 1. Identifier and basic information

Q1.1 ***Instruction:*** *The information collected below will not be linked to your confidential personal information. Please answer the questions below honestly. Your data will help us to understand the potential risk factors for SARS-CoV-2 infection among health workers and to prevent future infections of health workers.*

Q1.2 Gender

- Female  (1) 

- Male  (2) 

- Non-binary  (3) 

- Intersex  (4) 

- Other (specify)  (5) ________________________________________________

- Prefer not to answer  (6) 

Q1.3 Year of birth

▼ 2004 (4) ... 1920 (89)

|  |
| --- |

Q1.4 
Postal code
 (only the first 3 characters, e.g. V6T)

________________________________________________________________

| Page Break |  |
| --- | --- |

Q1.5 Status in Canada

- Citizen  (1) 

- Permanent resident  (2) 

- Visitor  (3) 

- Prefer not to answer  (4) 

Q1.6 Are you an Indigenous person?

- Yes  (1) 

- No  (2) 

- Prefer not to answer  (3) 

Q1.7 Race / ethnicity

- Asian  (1) 

- Black  (2) 

- Hispanic or Latinx  (3) 

- Non-Hispanic white  (4) 

- Other (specify)  (5) ________________________________________________

- Prefer not to answer  (6) 

| Page Break |  |
| --- | --- |

Q104 Did you work with the Vancouver Coastal Health Authority at any time between March 1, 2020 and the present day? (This could be as an employee, contractor, consultant, volunteer, etc.)

- Yes  (1) 

- No  (2) 

Q1.8 Occupation in health care facility (please select the option that most closely describes your occupation)

- Medical doctor  (1) 

- Registered nurse (or equivalent)  (2) 

- Assistant nurse, nurse technician (or equivalent)  (3) 

- Radiology / x-ray technician  (4) 

- Phlebotomist  (5) 

- Pharmacist  (6) 

- Physical therapist  (7) 

- Care aide  (8) 

- Nutritionist / dietician  (9) 

- Laboratory personnel  (10) 

- Admission / reception clerk  (11) 

- Patient transporter  (12) 

- Catering staff  (13) 

- Cleaner  (14) 

- Administration / clerk  (15) 

- Other (specify)  (16) ________________________________________________

Q1.9 Work setting

- Acute care  (1) 

- Community  (2) 

- Long-term care  (3) 

Q1.10 Education level

- None  (1) 

- Primary  (2) 

- Secondary  (3) 

- Tertiary / University  (4) 

- Postgraduate  (5) 

- Prefer not to answer  (6) 

End of Block: 1. Identifier and basic information

Start of Block: 2. Testing context

|  |  |
| --- | --- |

Q2.1 COVID-19 test date

*If you have taken the COVID-19 test more than once, please provide the date for the test for which you have the clearest recall of your activities in the 2 weeks before it.  If you are unsure of the date, please use the best estimate possible.*

(yyyy/mm/dd)

________________________________________________________________

| Page Break |  |
| --- | --- |

Q2.2 Does your job involve direct care for COVID-19 patients?

- Yes  (1) 

- No  (2) 

Display This Question:

If Does your job involve direct care for COVID-19 patients? = Yes

Q2.3 Please specify the number of days dedicated to COVID-19 patients only during the 2 weeks before your test date:

▼ 0 (1) ... 14 (15)

Q2.4 In the 2 weeks before your test, what section of the health facility did you spend the most working hours in?  (Select only one)

- Emergency room  (1) 

- Outpatient clinic  (2) 

- Inpatient ward / room  (3) 

- Intensive care  (4) 

- NICU  (5) 

- Theatre  (6) 

- Lab  (7) 

- Radiology  (8) 

- Reception / triage  (9) 

- Security  (10) 

- Non-patient access areas  (11) 

- Other (specify)  (12) ________________________________________________

| Page Break |  |
| --- | --- |

Q2.5 Outside of your occupational duties, were you in extended close contact (within 2 meters for 15 minutes or more) with a person or persons **known to have been diagnosed with COVID-19** in the 14 days before your test?

- Yes  (1) 

- No  (2) 

- Not sure / unaware  (3) 

Display This Question:

If Outside of your occupational duties, were you in extended close contact (within 2 meters for 15 m... = Yes

Q2.6 
Was this contact:

*(Check all that apply)*

- Household contact: school-age child  (1) 

- Household contact: other household member  (5) 

- Professional colleague(s)  (2) 

- Everyday social interactions (e.g. school, religious event, public transport, restaurant, grocery store, café)  (3) 

- Other (specify)  (4) ________________________________________________

Display This Question:

If Outside of your occupational duties, were you in extended close contact (within 2 meters for 15 m... = Yes

Q2.7 How long was this contact?

- Most days (≥ 8 days)  (1) 

- Some days (4 - 7 days)  (2) 

- Few days (1 - 3 days)  (3) 

- Less than a day  (4) 

| Page Break |  |
| --- | --- |

Q2.8 Outside of your occupational duties, were you in extended close contact (within 2 meter for 15 minutes or more) with a person or persons with **symptoms related to COVID-19** in the 2 weeks before your test?
Symptoms may include fever, cough, runny nose, sore throat, shortness of breath, etc.

- Yes  (1) 

- No  (2) 

- Not sure  (3) 

Display This Question:

If Outside of your occupational duties, were you in extended close contact (within 2 meter for 15 mi... = Yes

Q2.9 
Was this contact:

*(Check all that apply)*

- Household contact: school-age child  (1) 

- Household contact: other household member  (5) 

- Professional colleague(s)  (2) 

- Everyday social interactions (e.g. school, religious event, public transport, restaurant, grocery store, café)  (3) 

- Other (specify)  (4) ________________________________________________

Display This Question:

If Outside of your occupational duties, were you in extended close contact (within 2 meter for 15 mi... = Yes

Q2.10 How long was this contact?

- Most days (≥ 8 days)  (1) 

- Some days (4 - 7 days)  (2) 

- Few days (1 - 3 days)  (3) 

- Less than a day  (4) 

| Page Break |  |
| --- | --- |

Q2.11 
In the 2 weeks before your test, did you return from international travel?

- Yes  (1) 

- No  (2) 

Display This Question:

If In the 2 weeks before your test, did you return from international travel? = Yes

Q2.12 Which country?

________________________________________________________________

| Page Break |  |
| --- | --- |

Q2.13 
In the 2 weeks before your test, how often did you use public transport?

- Most days (≥ 8 days)  (1) 

- Some days (4 - 7 days)  (2) 

- Few days (≤ 3 days)  (3) 

- Did not use public transport  (4) 

Display This Question:

If In the 2 weeks before your test, how often did you use public transport? != Did not use public transport

Q2.14 What means of public transport did you use most frequently in that period? (Select only one)

- Train  (1) 

- Bus  (2) 

- Taxi or ride hailing (regular taxi, Uber, Lyft, etc.)  (3) 

- Ferry  (4) 

- Car share (Evo, Modo, etc.)  (5) 

- Carpool  (6) 

- Bike share  (8) 

- Mix of the above means  (9) 

Display This Question:

If In the 2 weeks before your test, how often did you use public transport? != Did not use public transport

Q2.15 
Whilst in public transport, what precautions did you utilize?

*(Check all that apply)*

- Face mask  (1) 

- Physical distancing, when possible  (2) 

- ⊗None  (3) 

Display This Question:

If Whilst in public transport, what precautions did you utilize? (Check all that apply) = Face mask

Q2.16 What type of face mask did you use most frequently on public transport?

- N95 or equivalent  (1) 

- Surgical mask  (2) 

- Cloth/fabric mask  (3) 

- Other (specify)  (4) ________________________________________________

| Page Break |  |
| --- | --- |

Q2.17 In the 2 weeks before your test, how often did
you have social interaction with individuals outside of work, home or transport
(e.g. in bars, clubs, shops, parties, religious events, funerals etc.)?

- Most days (≥ 8 days)  (1) 

- Some days (4 - 7 days)  (2) 

- Few days (≤ 3 days)  (3) 

- Did not have any such social interactions  (4) 

Display This Question:

If In the 2 weeks before your test, how often did you have social interaction with individuals outsi... != Did not have any such social interactions

Q2.18 Which of these applied most to the social interaction(s)?

- Activity was held outdoors  (1) 

- Activity was held indoors  (2) 

- Mix of both  (3) 

Display This Question:

If In the 2 weeks before your test, how often did you have social interaction with individuals outsi... != Did not have any such social interactions

Q2.19 
Which of these precautions were taken during most social interaction(s)?

- Face masks and physical distancing  (1) 

- Face masks, but no physical distancing  (2) 

- Physical distancing, but no face masks  (3) 

- No masks or distancing precautions  (4) 

Display This Question:

If Which of these precautions were taken during most social interaction(s)? = Face masks and physical distancing

Or Which of these precautions were taken during most social interaction(s)? = Face masks, but no physical distancing

Q2.20 
What type of mask did you use most frequently?

- N95 or equivalent  (1) 

- Surgical mask  (2) 

- Cloth / fabric mask  (3) 

- Other (specify)  (4) ________________________________________________

Display This Question:

If In the 2 weeks before your test, how often did you have social interaction with individuals outsi... != Did not have any such social interactions

Q2.21 Approximately how many people outside your household contacts were present at the largest gathering you attended?

- Less than 5  (1) 

- 5 - 15  (2) 

- 15 - 30  (3) 

- 31 - 50  (4) 

- More than 50  (5) 

| Page Break |  |
| --- | --- |

Q2.22 In the 2 weeks before your test, were you in extended close contact (within 2 metres for 15 minutes or more) with co-workers **at work**?  This includes contact in break/coffee rooms.

- Yes  (1) 

- No  (2) 

Display This Question:

If In the 2 weeks before your test, were you in extended close contact (within 2 metres for 15 minut... = Yes

Q2.23 How frequently were you in extended close contact with co-workers at work?

- Most days (≥ 8 days)  (1) 

- Some days (4 - 7 days)  (2) 

- Few days (1 - 3 days)  (3) 

- Less than a day  (4) 

Display This Question:

If In the 2 weeks before your test, were you in extended close contact (within 2 metres for 15 minut... = Yes

|  |
| --- |

Q2.24 Please tick the box that best describes how OFTEN
you used personal protective equipment (PPE) while in close contact with
coworkers in the 2 weeks before your test

|  | Always (1) | Most of the time (2) | Occasionally (3) | Rarely (4) | Never (5) |
| --- | --- | --- | --- | --- | --- |
| Medical / surgical masks (1) |  |  |  |  |  |
| Respirator (N95, FFP2 or equivalent) (2) |  |  |  |  |  |
| Face shield or goggles / glasses (3) |  |  |  |  |  |
| Gloves (4) |  |  |  |  |  |
| Gowns and coveralls (5) |  |  |  |  |  |
| Head cover (6) |  |  |  |  |  |
| Shoe covers (7) |  |  |  |  |  |
| Other (optional; specify) (8) |  |  |  |  |  |

Display This Question:

If In the 2 weeks before your test, were you in extended close contact (within 2 metres for 15 minut... = Yes

Q2.25 Were you made aware that a coworker you may
have come in contact with at work tested positive for COVID-19 in the 2 weeks
before your test?

- Yes  (1) 

- No  (2) 

| Page Break |  |
| --- | --- |

Q84 Does your job involve contact with patients, their materials or belongings, or medical equipment that patients may have come in contact with?

- Yes  (1) 

- No  (2) 

| Page Break |  |
| --- | --- |

End of Block: 2. Testing context

Start of Block: 3. Adherence to IPC

Display This Question:

If Does your job involve contact with patients, their materials or belongings, or medical equipment... = Yes

Q3.1 **Adherence to infection prevention and control (IPC) measures**

*(except otherwise stated, these questions refer to the 2 weeks before your test date)*

Display This Question:

If Does your job involve contact with patients, their materials or belongings, or medical equipment... = Yes

Q3.2 In the two weeks before your test, did you know what routine (standard) IPC practices were?

- Yes  (1) 

- No  (2) 

- Not sure / can't remember  (3) 

Display This Question:

If Does your job involve contact with patients, their materials or belongings, or medical equipment... = Yes

Q3.3 Did you receive any IPC training in the three
months before your test?

- Yes  (1) 

- No  (2) 

- Not sure / can't remember  (3) 

Display This Question:

If Did you receive any IPC training in the three months before your test? = Yes

And Does your job involve contact with patients, their materials or belongings, or medical equipment... = Yes

|  |
| --- |

Q3.4 What was the date of
your last IPC training?

- I know the exact date  *(yyyy/mm/dd)*  (1) ________________________________________________

- Uncertain of the exact date, but less than 1 month before  (2) 

- Uncertain of the exact date, but more than 1 month before  (3) 

Display This Question:

If Did you receive any IPC training in the three months before your test? = Yes

And Does your job involve contact with patients, their materials or belongings, or medical equipment... = Yes

Q3.5 In the three month before your test date, how
much cumulative IPC training (routine practices and additional
precautions) did you receive?

- Less than 2 hours  (1) 

- More than 2 hours  (2) 

Display This Question:

If Did you receive any IPC training in the three months before your test? = Yes

And Does your job involve contact with patients, their materials or belongings, or medical equipment... = Yes

Q3.6 Was the IPC training on personal protective
equipment (PPE) carried out remotely (e.g. presentations only, e-learning) or were practical sessions on standard precautions/additional precautions conducted?

- Only remotely / online  (1) 

- Only practical  (2) 

- Both  (3) 

| Page Break |  |
| --- | --- |

Display This Question:

If Does your job involve contact with patients, their materials or belongings, or medical equipment... = Yes

Q3.7 Before your COVID-19 test, did you know the recommended
moments for hand hygiene in health care?

- Yes  (1) 

- No  (2) 

- Not sure / can't remember  (3) 

Display This Question:

If Before your COVID-19 test, did you know the recommended moments for hand hygiene in health care? = Yes

And Does your job involve contact with patients, their materials or belongings, or medical equipment... = Yes

Q3.8 Did you follow these recommended hand hygiene
practices?

- Always  (1) 

- Most of the time  (2) 

- Occasionally  (3) 

- Rarely  (4) 

- Never  (5) 

| Page Break |  |
| --- | --- |

Display This Question:

If Does your job involve contact with patients, their materials or belongings, or medical equipment... = Yes

Q3.9 Before your test date, were you using alcohol-based
hand rub or soap and water:

|  | Always (1) | Most of the time (2) | Occasionally (3) | Rarely (4) | Never (5) |
| --- | --- | --- | --- | --- | --- |
| Before touching a patient? (1) |  |  |  |  |  |
| Before cleaning/aseptic procedures? (2) |  |  |  |  |  |
| After (risk of) body fluid exposure? (3) |  |  |  |  |  |
| After touching a patient? (4) |  |  |  |  |  |
| After touching a patient’s surroundings? (5) |  |  |  |  |  |

Display This Question:

If Does your job involve contact with patients, their materials or belongings, or medical equipment... = Yes

Q3.10 Was alcohol-based hand rub available at point of
care?

- Yes  (1) 

- No  (2) 

- Occasionally  (3) 

- Not sure  (4) 

Display This Question:

If Does your job involve contact with patients, their materials or belongings, or medical equipment... = Yes

|  |
| --- |

Q3.11 Please tick the box that best describes how you
OFTEN used personal protective equipment (PPE) while encountering patients in
the 2 weeks before your test.

|  | Always (1) | Most of the time (2) | Occasionally (3) | Rarely (4) | Never (5) |
| --- | --- | --- | --- | --- | --- |
| Medical / surgical masks (1) |  |  |  |  |  |
| Respirator (N95, FFP2 or equivalent) (2) |  |  |  |  |  |
| Face shield or goggles / glasses (3) |  |  |  |  |  |
| Gloves (4) |  |  |  |  |  |
| Gowns and coverall (5) |  |  |  |  |  |
| Head cover (6) |  |  |  |  |  |
| Shoe covers (7) |  |  |  |  |  |
| Other (optional; specify) (8) |  |  |  |  |  |

Display This Question:

If Does your job involve contact with patients, their materials or belongings, or medical equipment... = Yes

Q3.12 Did you experience difficulty getting PPE in the
2 weeks before your test?

- Yes  (1) 

- No  (2) 

Display This Question:

If Did you experience difficulty getting PPE in the 2 weeks before your test? = Yes

And Does your job involve contact with patients, their materials or belongings, or medical equipment... = Yes

Q3.13 Which PPE did you experience difficulty getting? (Select all that apply)

- Medical / surgical masks  (1) 

- Face shields or goggles / glasses  (2) 

- Gloves  (3) 

- Gowns or coveralls  (4) 

- Head covers  (5) 

- Respirators (e.g. N95, FFP2 or equivalent)  (6) 

- Shoe covers  (7) 

- Other (specify)  (8) ________________________________________________

Display This Question:

If Does your job involve contact with patients, their materials or belongings, or medical equipment... = Yes

Q3.14 Did you reuse any PPE in the 2 weeks before your
test on account of an inadequate supply?

- Yes  (1) 

- No  (2) 

Display This Question:

If Did you reuse any PPE in the 2 weeks before your test on account of an inadequate supply? = Yes

And Does your job involve contact with patients, their materials or belongings, or medical equipment... = Yes

Q3.15 Which PPE did you reuse? (Select all that apply)

- Medical / surgical masks  (1) 

- Face shields or goggles / glasses  (2) 

- Gloves  (3) 

- Gowns or coveralls  (4) 

- Head covers  (5) 

- Respirators (e.g. N95, FFP2 or equivalent)  (6) 

- Shoe covers  (7) 

- Other (specify)  (8) ________________________________________________

| Page Break |  |
| --- | --- |

End of Block: 3. Adherence to IPC

Start of Block: 4. Exposures to COVID patients

Display This Question:

If Does your job involve contact with patients, their materials or belongings, or medical equipment... = Yes

Q4.1 In relation to your test date, when was your
earliest exposure to a confirmed COVID-19 patient at work?

- 3 days or less before the test  (1) 

- 4 - 7 days before the test  (2) 

- 8 or more days before the test  (3) 

- I was not exposed to a known COVID-19 patient  (4) 

Display This Question:

If In relation to your test date, when was your earliest exposure to a confirmed COVID-19 patient at... != I was not exposed to a known COVID-19 patient

And Does your job involve contact with patients, their materials or belongings, or medical equipment... = Yes

|  |
| --- |

Q4.2 
Before your test date, how many COVID-19 patients were you exposed to during your occupational duties? 

*(An exact number or an estimated range is permissible)*

________________________________________________________________

Display This Question:

If In relation to your test date, when was your earliest exposure to a confirmed COVID-19 patient at... != I was not exposed to a known COVID-19 patient

And Does your job involve contact with patients, their materials or belongings, or medical equipment... = Yes

Q4.3 Did you have close contact (within 2 metres) with
the COVID-19 patient(s) since their admission?

- Yes  (1) 

- No  (2) 

- Unknown  (3) 

Display This Question:

If Did you have close contact (within 2 metres) with the COVID-19 patient(s) since their admission? = Yes

And Does your job involve contact with patients, their materials or belongings, or medical equipment... = Yes

Q4.4 How many times (total) did you have close contact with the COVID-19 patient(s)?

- < 10 times  (1) 

- 10 - 50 times  (2) 

- > 50 times  (3) 

Display This Question:

If Did you have close contact (within 2 metres) with the COVID-19 patient(s) since their admission? = Yes

And Does your job involve contact with patients, their materials or belongings, or medical equipment... = Yes

|  |
| --- |

Q4.5 Please specify exactly how many times, if you can recall:

*(optional)*

________________________________________________________________

Display This Question:

If Did you have close contact (within 2 metres) with the COVID-19 patient(s) since their admission? = Yes

And Does your job involve contact with patients, their materials or belongings, or medical equipment... = Yes

Q4.6 What was the duration of the longest of the close contacts?

- Less than 15 minutes  (1) 

- More than 15 minutes  (2) 

- Unknown  (3) 

Display This Question:

If In relation to your test date, when was your earliest exposure to a confirmed COVID-19 patient at... != I was not exposed to a known COVID-19 patient

And Does your job involve contact with patients, their materials or belongings, or medical equipment... = Yes

Q4.7 Were you present for any aerosolizing procedures on a COVID-19 patient?

- Yes  (1) 

- No  (2) 

- Unknown  (3) 

Display This Question:

If Were you present for any aerosolizing procedures on a COVID-19 patient? = Yes

And Does your job involve contact with patients, their materials or belongings, or medical equipment... = Yes

Q4.8 
Which procedure(s)?

*(Check all that apply)*

- Tracheal intubation  (4) 

- Nebulizer treatment  (7) 

- Open airway suctioning  (8) 

- Collection of sputum  (9) 

- Tracheostomy  (10) 

- Bronchoscopy  (11) 

- Cardiopulmonary resuscitation (CPR)  (12) 

- Other (specify)  (13) ________________________________________________

Display This Question:

If Were you present for any aerosolizing procedures on a COVID-19 patient? = Yes

And Does your job involve contact with patients, their materials or belongings, or medical equipment... = Yes

Q4.9 Did you wear PPE during the aerosolizing procedures?

- Yes  (1) 

- No  (2) 

- Unknown  (3) 

Display This Question:

If Did you wear PPE during the aerosolizing procedures? = Yes

And Does your job involve contact with patients, their materials or belongings, or medical equipment... = Yes

Q4.10 
What PPE was worn during the aerosolizing procedures?

*(Tick all that apply)*

- Medical / surgical masks  (1) 

- Respirators (e.g. N95, FFP2 or equivalent)  (2) 

- Face shields or goggles / glasses  (3) 

- Gloves  (4) 

- Gowns or coveralls  (5) 

- Head covers  (6) 

- Shoe covers  (7) 

- Other (specify)  (8) ________________________________________________

| Page Break |  |
| --- | --- |

Display This Question:

If Does your job involve contact with patients, their materials or belongings, or medical equipment... = Yes

Q4.11 If you wore a respirator at work in the 2 weeks before your COVID-19 test, was it fit tested?

- Yes  (1) 

- No  (2) 

- Unknown  (3) 

- I did not wear a respirator in the 2 weeks before the COVID-19 test  (4) 

Display This Question:

If If you wore a respirator at work in the 2 weeks before your COVID-19 test, was it fit tested? = Yes

And Does your job involve contact with patients, their materials or belongings, or medical equipment... = Yes

Q4.12 When was the fit testing done?

- Within 2 years of your test date  (1) 

- More than 2 years before your test date  (2) 

- Don't know  (3) 

| Page Break |  |
| --- | --- |

Display This Question:

If Does your job involve contact with patients, their materials or belongings, or medical equipment... = Yes

Q4.13 Did you have direct contact with the **patient’s materials** in the 2 weeks before your test?

(Patient’s materials include personal belongings, linen and medical equipment that the patient may have come into contact with)

- Yes  (1) 

- No  (2) 

- Unknown  (3) 

Display This Question:

If Did you have direct contact with the patient’s materials in the 2 weeks before your test? (Patien... = Yes

And Does your job involve contact with patients, their materials or belongings, or medical equipment... = Yes

Q4.14 Which patient materials did you have direct contact with? Tick all that apply:

- Personal items  (2) 

- Linens / clothes  (3) 

- Medical devices used on the patient  (4) 

- Medical equipment connected to the patient (e.g. ventilator, infusion pump, etc.)  (5) 

- Other (specify)  (6) ________________________________________________

Display This Question:

If Did you have direct contact with the patient’s materials in the 2 weeks before your test? (Patien... = Yes

And Does your job involve contact with patients, their materials or belongings, or medical equipment... = Yes

Q4.15 How many times during the period did you have direct contact with the patient's materials (total)?

- < 10 times  (1) 

- 10 - 50 times  (2) 

- > 50 times  (3) 

Display This Question:

If Did you have direct contact with the patient’s materials in the 2 weeks before your test? (Patien... = Yes

And Does your job involve contact with patients, their materials or belongings, or medical equipment... = Yes

|  |
| --- |

Q4.16 Please
  specify exactly how many times, if you can
  recall:(optional)

________________________________________________________________

Display This Question:

If Did you have direct contact with the patient’s materials in the 2 weeks before your test? (Patien... = Yes

And Does your job involve contact with patients, their materials or belongings, or medical equipment... = Yes

Q4.17 Did you come into contact with the patient's body fluids via the patient's materials?

- Yes  (1) 

- No  (2) 

- Unknown  (3) 

Display This Question:

If Did you come into contact with the patient's body fluids via the patient's materials? = Yes

And Does your job involve contact with patients, their materials or belongings, or medical equipment... = Yes

Q4.18 Which body fluids:

- Blood  (13) 

- Vomit  (12) 

- Urine  (11) 

- Stool  (7) 

- Mucus from the nose or lungs  (8) 

- Sweat  (9) 

- Breast milk  (4) 

- Other (specify)  (14) ________________________________________________

Display This Question:

If Did you have direct contact with the patient’s materials in the 2 weeks before your test? (Patien... = Yes

And Does your job involve contact with patients, their materials or belongings, or medical equipment... = Yes

|  |
| --- |

Q4.19 Please select which most accurately apply to
your PPE while handling patient’s materials:

|  | Always (1) | Most of the time (2) | Occasionally (3) | Rarely (4) | Never (5) |
| --- | --- | --- | --- | --- | --- |
| Medical / surgical masks (1) |  |  |  |  |  |
| Respirator (N95, FFP2 or equivalent) (2) |  |  |  |  |  |
| Face shield or goggles / glasses (3) |  |  |  |  |  |
| Gloves (4) |  |  |  |  |  |
| Gowns and coverall (5) |  |  |  |  |  |
| Head cover (6) |  |  |  |  |  |
| Shoe covers (7) |  |  |  |  |  |
| Other (optional; specify) (8) |  |  |  |  |  |

| Page Break |  |
| --- | --- |

Display This Question:

If Does your job involve contact with patients, their materials or belongings, or medical equipment... = Yes

Q4.20 Did you have direct contact with the surfaces around the patient?

- Yes  (1) 

- No  (2) 

- Unknown  (3) 

Display This Question:

If Did you have direct contact with the surfaces around the patient? = Yes

And Does your job involve contact with patients, their materials or belongings, or medical equipment... = Yes

Q4.21 Which surfaces did you have direct contact with?

- Bed  (1) 

- Bathroom  (2) 

- Ward corridor  (3) 

- Patient table  (4) 

- Bedside table  (5) 

- Dining table  (6) 

- Medical gas panel  (7) 

- Other (specify)  (8) ________________________________________________

Display This Question:

If Did you have direct contact with the surfaces around the patient? = Yes

And Does your job involve contact with patients, their materials or belongings, or medical equipment... = Yes

Q4.22 Approximately how many times in the 2 weeks before your test did you make such contact in total?

- < 10 times  (1) 

- 10 - 50 times  (2) 

- > 50 times  (3) 

Display This Question:

If Did you have direct contact with the surfaces around the patient? = Yes

And Does your job involve contact with patients, their materials or belongings, or medical equipment... = Yes

|  |
| --- |

Q4.23 Please
  specify exactly how many times, if you can
  recall:(optional)

________________________________________________________________

Display This Question:

If Did you have direct contact with the surfaces around the patient? = Yes

And Does your job involve contact with patients, their materials or belongings, or medical equipment... = Yes

|  |
| --- |

Q4.24 Please select which most accurately describes your PPE use while making
  direct contact with the surfaces around the patient

|  | Always (1) | Most of the time (2) | Occasionally (3) | Rarely (4) | Never (5) |
| --- | --- | --- | --- | --- | --- |
| Medical / surgical masks (1) |  |  |  |  |  |
| Respirator (N95, FFP2 or equivalent) (2) |  |  |  |  |  |
| Face shield or goggles / glasses (3) |  |  |  |  |  |
| Gloves (4) |  |  |  |  |  |
| Gowns and coverall (5) |  |  |  |  |  |
| Head cover (6) |  |  |  |  |  |
| Shoe covers (7) |  |  |  |  |  |
| Other (optional; specify) (8) |  |  |  |  |  |

End of Block: 4. Exposures to COVID patients

Start of Block: 5. Health worker pre-existing conditions

Q5.1 Before your test date, did you have any
underlying disease or pre-existing condition(s)?

- Pregnancy  (1) 

- Obesity (optional: specify BMI)  (2) ________________________________________________

- Cancer  (3) 

- Diabetes  (4) 

- HIV / other immune deficiency  (5) 

- Heart disease  (6) 

- Asthma (requiring medication)  (7) 

- Chronic lung disease (non-asthma)  (8) 

- Chronic liver disease  (9) 

- Chronic haematological disorder  (10) 

- Chronic kidney disease  (11) 

- Chronic neurological impairment / disease  (12) 

- Organ / bone marrow recipient  (13) 

- No  (14) 

- Unknown  (15) 

- Other (specify)  (16) ________________________________________________

Display This Question:

If Before your test date, did you have any underlying disease or pre-existing condition(s)? = Pregnancy

Q5.2 Specify trimester of pregnancy:

- First  (1) 

- Second  (2) 

- Third  (3) 

- Unknown  (4) 

Q5.3 What was your smoking status at the time of your test?

- Current smoker  (1) 

- Past smoker  (2) 

- Never smoked  (3) 

Q85 Have you received a COVID-19 vaccine?

- Yes - 2 doses received  (2) 

- Yes - 1 dose received  (1) 

- No  (3) 

Display This Question:

If Have you received a COVID-19 vaccine? != No

|  |  |
| --- | --- |

Q86 
What was the date of the **first dose**?

(yyyy/mm/dd)

________________________________________________________________

| Page Break |  |
| --- | --- |

Display This Question:

If Have you received a COVID-19 vaccine? = Yes - 2 doses received

|  |  |
| --- | --- |

Q87 
What was the date of the **second dose**?

(yyyy/mm/dd)

________________________________________________________________

| Page Break |  |
| --- | --- |

Q5.4 Thank
you for completing the questionnaire. You may submit your responses now or
proceed to answer a few short questions on your experience with COVID-19 testing and how you feel regarding safety from COVID-19.

- Submit now  (1) 

- Continue to additional questions  (2) 

End of Block: 5. Health worker pre-existing conditions

Start of Block: 6. COVID-19 Expereince

Q6.1  How satisfied were you with:

|  | Very dissatisfied (1) | Somewhat dissatisfied (2) | Neither satisfied nor dissatisfied (3) | Somewhat satisfied (4) | Very satisfied (5) |
| --- | --- | --- | --- | --- | --- |
| how quickly you could get tested? (1) |  |  |  |  |  |
| how quickly you received your result? (2) |  |  |  |  |  |
| instructions / communication you received related to getting tested for COVID-19? (3) |  |  |  |  |  |

Q6.2 
Any comments you want to share regarding your test experience or anything else?

*(optional)*

________________________________________________________________

________________________________________________________________

________________________________________________________________

________________________________________________________________

________________________________________________________________

| Page Break |  |
| --- | --- |

Q6.3 How would you describe how safe you feel regarding:

|  | Very unsafe (1) | Somewhat unsafe (2) | Neither safe nor unsafe (3) | Somewhat safe (4) | Very safe (5) |
| --- | --- | --- | --- | --- | --- |
| contracting COVID-19 at work (1) |  |  |  |  |  |
| contracting COVID-19 in the community (2) |  |  |  |  |  |

Q6.4 
Any comments you want to share regarding your safety from COVID-19 or anything else?

*(optional)*

________________________________________________________________

________________________________________________________________

________________________________________________________________

________________________________________________________________

________________________________________________________________

End of Block: 6. COVID-19 Expereince
